# Métastase anale d'un adénocarcinome du sigmoïde: la partie visible de l'iceberg

**DOI:** 10.11604/pamj.2014.18.177.3659

**Published:** 2014-06-24

**Authors:** Rachid El Barni, Mohamed Lahkim, Jawad Fassi Fihri, Abdelhadi Mejdane, Rachid Bouchama, Abdessamad Achour

**Affiliations:** 1 Service de chirurgie générale. Hôpital militaire Avicenne Marrakech, Maroc

**Keywords:** Adénocarcinome du sigmoïde, Métastase anale, adenocarcinoma of the sigmoid, aenal metastasis

## Abstract

Les auteurs rapportent l'observation d'une métastase anale d'un adénocarcinome sigmoïdien dont le traitement a consisté en une amputation abdominopérinéale du rectum, étendue au côlon sigmoïde avec colostomie iliaque gauche définitive. Les différents mécanismes d'extension tumorale sont discutés et la greffe de cellules tumorales viables exfoliées dans la lumière colique est le mécanisme le plus souvent retenu.

## Introduction

Le cancer anal est généralement un carcinome épidermoïde. La révélation d'un adénocarcinome colique par une fistule anale métastatique est une situation rare dont la gestion reste controversée. Nous rapportons une métastase d'un adénocarcinome sigmoïdien dans une fistule anale préexistante traitée par une amputation abdominopérinéale du rectum, étendue au côlon sigmoïde avec colostomie iliaque gauche définitive.

## Patient et observation

**Figure 1:**
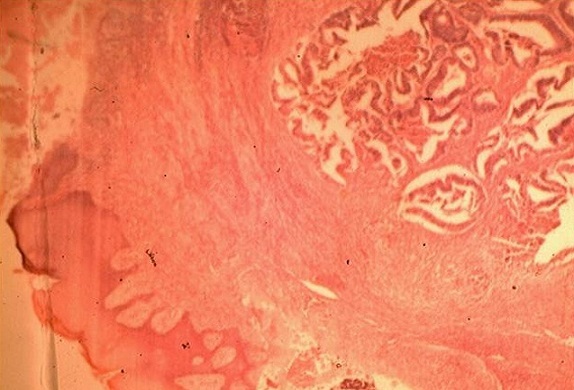
Histologie de la biopsie périnéale montrant l'envahissement cutané par un adénocarcinome lieberkuhnien moyennement différencié et infiltrant

**Figure 2:**
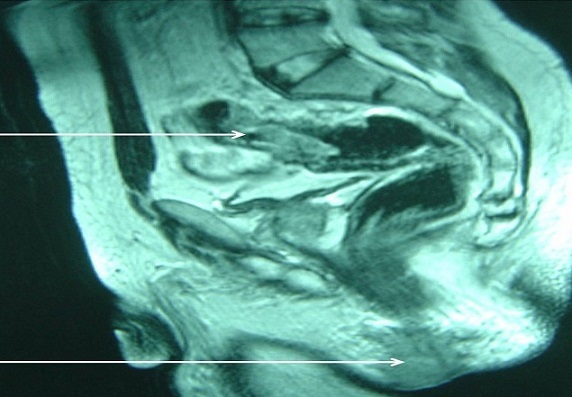
IRM pelvienne montrant un double processus lésionnel anal et sigmoïdien (flèches blanches)

**Figure 3:**
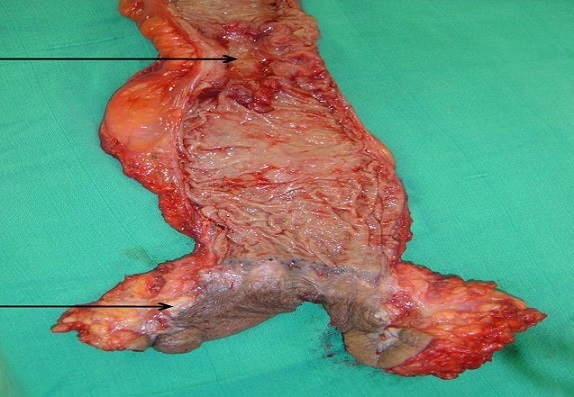
Pièce d'amputation abdominopérinéale montrant une double localisation néoplasique anale et sigmoïdienne (flèches noires)

O.H., âgé de 56 ans, était hospitalisé pour une fistule anale récidivante pour laquelle il était opéré il y a 15 ans. L'examen clinique trouvait une masse para-anale gauche dure et fistulisée avec écoulement purulent et sanglant. Une biopsie chirurgicale de cette masse était réalisée, et l’étude histologique de cette biopsie avait trouvé un envahissement cutané par un adénocarcinome lieberkuhnien moyennement différencié et infiltrant (Figure 1). Une coloscopie totale avait objectivé un processus ulcéro-bourgeonnant hémi-circonférentiel à 20 cm de la marge anale étendu sur 10 cm. L'examen anatomo-pathologique des biopsies coliques revenait en faveur d'un adénocarcinome lieberkuhnien moyennement différencié et infiltrant. Une imagerie par résonance magnétique du pelvis objectivait un processus lésionnel para-anal gauche de signal hétérogène bas en T1 et intermédiaire en T2. Ce processus était associé à un deuxième processus tissulaire sigmoïdien circonférentiel réduisant la lumière colique (Figure 2). Le bilan d'extension était négatif. Une amputation abdominopérinéale du rectum, étendue au côlon sigmoïde avec colostomie iliaque gauche définitive était réalisée (Figure 3). L'examen histologique de la pièce opératoire avait montrait un adénocarcinome colique moyennement différencié infiltrant Stade T3N1Mx p(TNM). La tranche de résection antérieure était occupée par un trajet fistuleux abritant des tubes néoplasiques. Une radio-chimiothérapie était administrée. Aucune récidive de la maladie n'avait été notée après 2 ans et 6 mois.

## Discussion

La découverte simultanée de deux tumeurs malignes de même type histologique pose un problème nosologique de leur filiation: tumeurs synchrones ou métastase [1]. Le premier cas de métastase de carcinome colorectal dans une fistule anale était décrit par Guiss [2] en 1954. Nous avons pu recenser dans la littérature 25 cas de métastase sur fistule anale préexistante (Tableau 1) [2–24]. Dans les métastases anales d'adénocarcinomes du sigmoïde, au sein ou non d'une suppuration [2, 3, 5, 17, 25, 26], il est possible d’évoquer: une extension intra-murale rétrograde de proche en proche, une invasion lymphatique rétrograde, une dissémination par voie veineuse rétrograde; mécanismes à ne retenir, en principe, que si les deux tumeurs ne sont pas “éloignées” l'une de l'autre [25, 27]. La greffe cellulaire tumorale par exfoliation cellulaire constitue en fait l'hypothèse la plus souvent retenue dans la littérature [3, 5, 27–30]. L'implantation de cellules tumorales au niveau du canal anal se fait habituellement sur une lésion préexistante telle qu'une fistule anale, une fissure, une cicatrice chirurgicale, ou une érosion muqueuse d'origine traumatique [1]. Le traitement des métastases anales des cancers sigmoïdiens repose essentiellement sur la chirurgie. Le choix est théoriquement à faire entre une excision de la tumeur anale en cas de lésion superficielle chez des patients âgés ou fragiles [26, 29, 31] et une amputation abdominopérinéale, traitement radical destiné à éviter les récidives locorégionales [32, 33]. La radiothérapie adjuvante isolée ou associée à la chimiothérapie peut être proposée en complément de la chirurgie, malgré l'absence de preuve de leur efficacité [33].

**Tableau 1:** Cas rapportés de métastases d'un adénocarcinome colorectal dans une fistule anale.

Auteur	Année	Age	Sexe	Siège de la tumeur primaire	Durée de la fistule anale	Dukes	Différenciation	Intervention	Recul sans récidive
**Guiss A**[2]	1954	47	M	Sigmoïde	2 mois	A	Moyenne	AAP	1 an 2 mois
**Killingback et al**. [3]	1965	63	M	Sigmoïde	8 ans	A	Bonne	AAP	-
**Parnes**[4]	1976	47	M	Sigmoïde	3 mois	B	Bonne	AAP	18 mois
**Rollinson and Dundas**[5]	1984	65	M	Recto- sigmoïde	20 ans	-	Bonne	AAP	10 mois
**Ueta et al**. [6]	1991	66	F	Sigmoïde	44 ans	B	Bonne	AAP	6 mois
**Thomas and Thompson**[7]	1992	68	M	Sigmoïde	1 an	B	Moyenne	AAP	-
**Tohira et al. (Japenese)**[8]	1998	75	M	Haut rectum	40 ans	B	Bonne	AAP	1 an
**Isbister**[9]	2000	47	M	Recto- sigmoïde	2 ans	C	Moyenne	-	-
**Isbister**[9]	2000	39	M	Sigmoïde	1 an	-	Bonne	RL	-
**Tokuhara et al**. [10]	2001	69	M	Sigmoïde	5 ans	B	Moyenne	AAP	1 an
**Yoshimura et al**. [11]	2001	59	M	Recto- sigmoïde	29 ans	C	Moyenne	AAP	3 ans 7 mois
**Shinohara et al**. [12]	2001	36	M	Moyen rectum	16 ans	C	Moyenne	RA + RL	6 mois
**Kouraklis et al**. [13]	2002	75	M	Sigmoïde	1 an	B	Moyenne	AAP	-
**Yagihashi et al**. [14]	2002	50	M	Sigmoïde	-	C	Bonne	EPT	3 ans 8 mois
**Shimoyama et al**. [15]	2003	61	M	Recto- sigmoïde	5 ans	C	Moyenne	AAP	5 ans
**Hyman and Kida**[16]	2003	66	M	Sigmoïde	15 ans	B	MoyenneCK7-/CK20 +	AAP	1 an
**Zbar and Shenoy**[17]	2004	72	M	Sigmoïde	4 ans	-	-	S + RL + Adj RCT	1 an 2 mois
**Gupta et al**. [18]	2005	44	M	Côlon gauche	-	C	Moyenne	HG + RL	3ans
**Hamada et al**. [19]	2005	53	M	Sigmoïde	7 ans	B	Bonne CK7-/CK20 +	RA + RL	1 an
**Ishiyama et al**. [20]	2006	53	M	Haut rectum	20 ans	C	Moyenne	RA + RL	10 mois Décès par carcinose
**Sandiford et al**. [21]	2006	72	M	Recto- sigmoïde	2 ans	B	Moyenne	S + RL + Adj RCT	14 mois
**Gravante et al**. [22]	2008	64	M	Côlon gauche	-	A	Moyenne CK7-/CK20 +	1-HG 2- AAP +Adj RCT	1 an
**Wakatsuki et al**. [23]	2008	57	M	Recto- sigmoïde	7 ans	B	Moyenne CK7-/CK20 +	1- RA 2- RL après 2 ans	3 ans 7 mois
**Benjelloun et al**. [24]	2012	68	M	Recto- sigmoïde	2 mois	B	Bonne CK7-/CK20 +	Néoadj. RCT RA + RL	3 ans
**Benjelloun et al**. [24]	2012	55	M	Recto- sigmoïde	10 ans	B	Bonne CK7-/CK20 +	Néoadj. RCT RA + RL	3 ans
**Notre cas**	2012	56	M	Sigmoïde	15 ans	C	Moyenne	AAP + Adj RCT	2 ans 6 mois

**AAP** : Amputation abdomino-périnéale. **RL** : Résection locale. **RA** : Résection antérieure. **EPT** : Exentération pelvienne totale. **S** : sigmoïdectomie. **HG** : Hémicolectomie gauche. **Adj** : Adjuvant. **Néoadj** : Néo-adjuvant. **RCT** : Radio-chimiothérapie.

## Conclusion

Il est nécessaire de pratiquer une biopsie devant toute fistule anale de présentation inhabituelle afin d’éliminer toute malignité qui peut être la partie visible de l'iceberg.
